# Exploring highly reliable substructures in auto-reconstructions of a neuron

**DOI:** 10.1186/s40708-021-00137-1

**Published:** 2021-08-24

**Authors:** Yishan He, Jiajin Huang, Gaowei Wu, Jian Yang

**Affiliations:** 1 Faculty of Information Technology, Beijing University of Technology, 100 Pingleyuan, Chaoyang District, Beijing, 100124 China; 2 Beijing International Collaboration Base On Brain Informatics and Wisdom Services, 100 Pingleyuan, Chaoyang District, Beijing, 100124 China; 3 School of Artificial Intelligence, University of Chinese Academy of Sciences, 19(A) Yuquan Road, Shijingshan District, Beijing, 100049 China; 4 Institute of Automation, Chinese Academy of Sciences, Haidian District, 95 Zhongguancun East Road, Beijing, 100190 China

**Keywords:** Neuronal morphology, Reconstruction, Local alignment, Motif

## Abstract

The digital reconstruction of a neuron is the most direct and effective way to investigate its morphology. Many automatic neuron tracing methods have been proposed, but without manual check it is difficult to know whether a reconstruction or which substructure in a reconstruction is accurate. For a neuron’s reconstructions generated by multiple automatic tracing methods with different principles or models, their common substructures are highly reliable and named individual motifs. In this work, we propose a Vaa3D-based method called Lamotif to explore individual motifs in automatic reconstructions of a neuron. Lamotif utilizes the local alignment algorithm in BlastNeuron to extract local alignment pairs between a specified objective reconstruction and multiple reference reconstructions, and combines these pairs to generate individual motifs on the objective reconstruction. The proposed Lamotif is evaluated on reconstructions of 163 multiple species neurons, which are generated by four state-of-the-art tracing methods. Experimental results show that individual motifs are almost on corresponding gold standard reconstructions and have much higher precision rate than objective reconstructions themselves. Furthermore, an objective reconstruction is mostly quite accurate if its individual motifs have high recall rate. Individual motifs contain common geometry substructures in multiple reconstructions, and can be used to select some accurate substructures from a reconstruction or some accurate reconstructions from automatic reconstruction dataset of different neurons.

## Introduction

The structure and function of neurons are very important for understanding the working mechanism of brains. Neuronal morphology is an important means to investigate neuronal structure and function. One major task of the US BRAIN Initiative (http://braininitiative.nih.gov/) and European Human Brain Project (https://www.humanbrainproject.eu/) [1] was to reconstruct and aggregate neuronal morphologies on scales up to the whole rodent brain. So far, researchers have invented many automatic tracing methods to efficiently generate a reconstruction of a neuron. Different tracing methods have different principles and/or models, and produce different reconstructions for a same neuron. However, a great proportion of the digitalized neurons so far was still acquired by manual tracing which is a highly labor intensive procedure. Without manual check, it is almost impossible to know whether a reconstruction or which substructure in a reconstruction is accurate enough for using with confidence. For a neuron’s reconstructions generated by some automatic tracing methods, their common substructures have a high degree of reliability, and can be used directly. We introduce a method to define and find these common substructures in a specified reconstruction and multiple reference reconstructions.

In recent years, many automatic methods and tools have been developed for digital reconstruction of neurons, such as automatic contour extraction [2], APP1 [3, 4], APP2 [5], MOST [6], SmartTracing [7], Ray casting [8], tTuFF [9], Rivulet [10], SparseTracer [11], M-AMST [12], Ensemble Neuron Tracer [13], Rivulet2 [14], FMST [15], MOST-based repairer [16], 3-D upgraded ray-shooting [17], and so on. Two memorabilia were held to promote the research of automatic tracing technologies. One is the DIADEM neuron reconstruction contest held in 2010 [18, 19], and another is the BigNeuron project [20] (http://bigneuron.org) launched in 2015. The BigNeuron project aims to gather a worldwide community to define and advance the state-of-the-art of single neuron reconstruction by benchtesting as many automatic neuron reconstruction methods as possible against as many neuron datasets as possible [20–22]. BigNeuron incorporated around 30 automatic tracing algorithms, which were implemented on a set of 30,000 + multi-dimensional neuronal image stacks and generated more than one million morphological reconstructions of neurons from different species (https://alleninstitute.org/bigneuron).

Automatic tracing methods are developed for different application scenarios and based on different models and/or principles, and typically have varying performance, especially on neuronal images with variable quality [20, 21]. Almost all automatic methods have not been directly cross-tested thoroughly, so it is unclear which methods are best matched with different imaging modalities or datasets [20]. Even the best tracing method is hard to make sure that its reconstruction is accurate everywhere on a neuron. One method might perform well at some substructures and another method might be good at other substructures. Reconstructions of a neuron generated by some different methods usually have some similar (or common) parts, which reflect a high degree of agreement reached by these methods. For a reconstruction, it is reasonable to suppose that its substructures common to many other reconstructions of the same neuron are accurate substructures, which are called individual motifs in this paper. In practice, in order to obtain morphological reconstructions with high-accuracy, neuronal reconstructions are often traced or checked segment by segment by human experts. Individual motifs can be taken as parts of a gold standard reconstruction without manual checking and is helpful for decreasing the workload of annotators.

Actually, common substructures in morphologies of different neurons are called motifs in computational biology. Wan et al. developed BlastNeuron to compare neurons in terms of their global appearance, detailed arborization patterns, and topological similarity [23]. The local alignment algorithm in BlastNeuron is capable of finding the corresponding branches or substructures in neuron morphologies of two tightly connected neurons, and pinpoints structure motifs of two similar neurons. Gillette et al. defined topological motifs in reconstructions of different neurons [24, 25]. The neuron topology was decomposed into sequences of branching patterns, and then a method was proposed to compare neuron structures using sequence alignment. The method is able to identify the difference in branching patterns in dendritic and axonic arbors, and extract common topological motifs in the structure [24, 25]. Topological motifs use the topology of neurons and look for common topological structures in different neurons. Individual motifs are different from above two kinds of motifs in definition and aim. Individual motifs are based on structure motifs in an objective reconstruction and many other reconstructions of a neuron, utilize the geometry of these reconstructions and explore common geometric substructures hidden in the objective and multiple other reference reconstructions.

In this work, we propose a method to find individual motifs in a specified objective reconstruction of a neuron, which makes use of local alignment in BlastNeuron and is called Lamotif. Lamotif consists of four main steps: pre-processing on automatically generated reconstructions, generating local alignment pairs using BlastNeuron (Basic Local Alignment Search Tool for Neurons) [23], constructing overlaps and obtaining individual motifs by some post-processing. Lamotif is implemented and tested on a reconstruction dataset of 163 neurons from different species. Experimental results show that individual motifs are almost on corresponding gold standard reconstruction and have much higher precision rate than these objective reconstructions themselves. Meanwhile, an objective reconstruction must be very accurate if the recall rate of its individual motifs is high (close to 1). Individual motifs are helpful for both selecting some accurate substructures from an automatic reconstruction or some accurate reconstructions from a dataset of neuronal reconstructions generated by an automatic tracing method.

The main contributions of this paper include the following:

- It proposes a method called Lamotif to explore common substructures in some reconstructions of a neuron generated by multiple automatic tracing methods, which makes use of local alignment in BlastNeuron.
- It introduces the idea ‘two heads are better than one’ to find some accurate substructures in an automatic reconstruction or some accurate reconstructions in a dataset of automatic reconstructions.
- It performs an experimental evaluation of Lamotif on reconstructions of 163 neurons from different species and analyzes its experimental results.

## Method

The overview of our proposed Lamotif and its main steps: pre-processing, generating local alignment pairs, constructing overlaps and post-processing overlaps are introduced in detail.

### Overview of Lamotif

For a neuron, many different reconstructions can be conveniently obtained by implementing various automatic tracing methods plugged in Vaa3D. The aim of Lamotif is to find morphological motifs in a specified objective reconstruction and multiple reference reconstructions. The objective reconstruction is a reconstruction generated by a relatively good automatic tracing algorithm, such as APP2 [4], Snake [26], Neutube [27] and NeuroGPS-Tree [28]. A reference reconstruction is the result of any other automatic tracing algorithm.

**Fig. 1:**
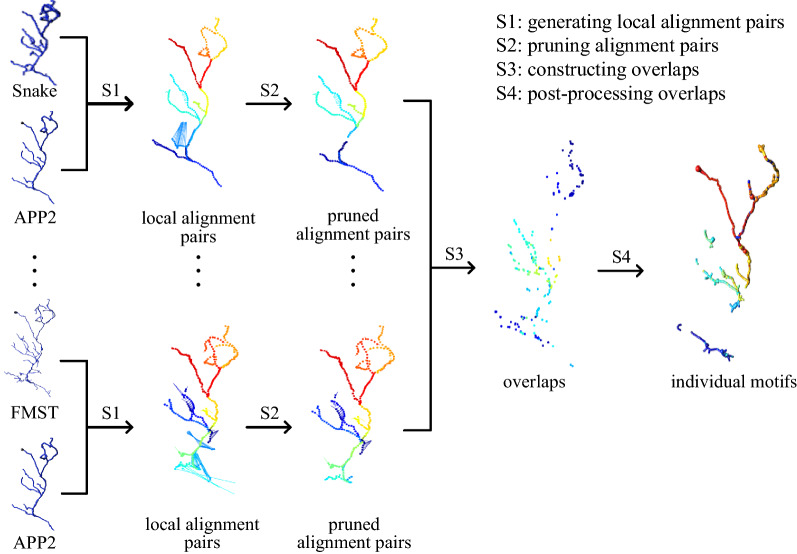
Overview of the proposed Lamotif method

The input of Lamotif is some automatically traced reconstructions represented in a SWC format [29], which describes a neuronal morphology as tree structures with location, node’s radius, parent node and some other attributes. The overview of Lamotif is demonstrated via an example (Fig. 1). Taking APP2 reconstruction as the objective reconstruction and some other reconstructions as references, Lamotif pre-processes each reconstruction, and constructs local alignment pairs between the objective and each reference by the local alignment algorithm in BlastNeuron [23] (step S1 in Fig. 1). Then, local alignment pairs are pruned (step S2 in Fig. 1) and overlapping nodes are selected (step S3 in Fig. 1). Finally, these nodes are connected into tree-like morphological structures and individual motifs are obtained after pruning some small fragments (step S4 in Fig. 1).

### Preprocessing reconstructions

Due to the diverse design of various tracing algorithms, their results might be quite different in the density of nodes, number of roots, positions of roots, and so on. It is necessary to implement some pre-processing steps to standardize all reconstructions and removing some obviously unreasonable reconstructions. Preprocessing steps contains resampling, sorting and filtering, and the former two can be carried out by running corresponding plugins in Vaa3D [30, 31]. All reconstructions are resampled with a fixed step length by running the resample_swc function. Then the number of nodes in each resampled reconstruction reflects the size of its neuronal tree. To satisfy the requirements of the local alignment algorithm, all reconstructions are reconnected into a single tree and the root node of each tree is reset to its node nearest to the root node of the objective reconstruction, which are actualized by implementing the sort function.

Reconstructions with too few or too many nodes do not make sense, and are excluded in subsequent steps. A bad reconstruction is picked out by comparing its node number to the average node number of all reconstructions. Let $n_{i}$ be the node number of the *i*th reconstruction, $n_{mean}$ and $n_{std}$ be the average and standard deviation of node numbers of all reconstructions. Only reconstructions satisfying the following condition are retained:

1 $$\left| {n_{i} - n_{mean} } \right| < k*n_{std} ,$$

where $k$ is parameter determined by users. It needs to be note that excluded reconstructions have much more or much less nodes while $k > 1$ (1.5 in our experiments), since $n_{std}$ is always quite big. The exclusion of bad reconstructions makes the subsequent processing steps more efficient.

### Generating local alignment pairs

The local alignment algorithm is the most important component of BlastNeuron [23]. The algorithm compares neuronal morphologies locally at the compartment level. It finds the corresponding relationship between segments of two reconstructions and constructs local alignment pairs accordingly by matching their topology and geometry. The algorithm is utilized to find local alignment pairs between the objective reconstruction and each reference reconstruction in Lamotif.

The inputs of local alignment algorithm are two reconstructions (denoted by $X$ and ${\text{Y}}$) with tree-topology structures. Both reconstructions are firstly normalized to the same center location in 3D space by using resampling and the PCA (principal component analysis) method, and a matrix of Euclidean distance from each node in ${\text{X}}$ to all other nodes in ${\text{Y}}$ is constructed. A RANSAC sampling process [32] is used to estimate the optimal affine transformation from ${\text{X}}$ to ${\text{Y}}$, then the correspondence relation between ${\text{X}}$ and ${\text{Y}}$ can be found in a same space. Considering the inhomogeneity of the shape and location of neuronal arbors, the algorithm decomposes both reconstructions into several simple line segments bounded by branching nodes and tip nodes. A dynamic programming algorithm is used to calculate distances between these segments and their inner nodes, and corresponding nodes are matched to provide local alignment pairs.

### Constructing overlaps

**Fig. 2:**
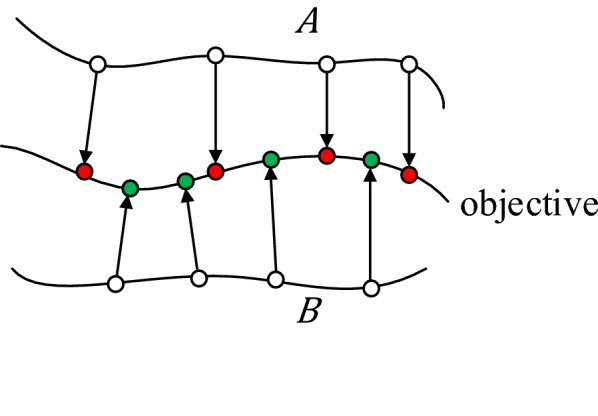
Representative nodes of local alignment pairs. Objective is the objective reconstruction, and $A$ and $B$ are two reference reconstructions. Unfilled circles are nodes on reference reconstructions, arrows represent local alignments between two nodes, and filled circles (red and green) are representative nodes and also candidates for the final overlap

Matched local alignment pairs consist of line segments between two nodes in the objective reconstruction and a reference reconstruction. The shorter a pair is, the better these two connected nodes match. Only pairs shorter than a given threshold (20 μm in our experiments) are selected to construct overlap and the node in the objective reconstruction is used to represent the pair. The distance between two local alignment pairs for two reference reconstructions $A$ and $B$ is defined by the Euclidean distance between their representative nodes on the objective reconstruction (red and green filled circles, respectively, in Fig. 2). If two representative nodes have distance less than a given neighbor distance threshold *d*, they are put into an overlapping set for these two reference reconstructions. If a representative node belongs to more than a given count number $c$ overlapping sets, it is defined to be a node in the final overlap between the objective and reference reconstructions.

### Post-processing overlaps

Though all points in the overlapping set are selected from nodes of the objective reconstruction, they might be quite sparse or discontinuous in the reconstruction. These points are processed to compose the skeleton of the final morphological structure by using the sort function in Vaa3D. The sorted tree-like structures may contain some very short trees with only one to two nodes, and short trees (with no more 3 nodes) are pruned.

## Experiment and its results

Lamotif is implemented on a reconstruction dataset of 163 neurons from different species, and experiment aims to evaluate its capability in discovering some accurate substructures in an automatic reconstruction and some accurate reconstructions in many reconstructions.

### Experiment dataset

The gold166 dataset was released by the BigNeuron project (https://alleninstitute.org/bigneuron), which consists of some benchtesting reconstructions of 163 neurons from multiple species (166 neurons except 3 neurons without gold standard reconstruction). These neurons are 8 neurons of chick, 2 neurons of frog, 91 neurons of fruitfly, 11 neurons of human, 31 neurons of mouse, 7 neurons of silkmoth and 13 neurons of zebrafish. For each neuron, the benchtesting reconstructions include a gold standard reconstruction and about 40 automatic reconstructions by 20 + automatic tracing algorithms with different parameters. APP2 [4], Snake [26], Neutube [27] and NeuroGPS-Tree [28] are four tracing algorithms with good performance and generate most reconstructions (the number of each algorithm is given in Table 1). Their reconstructions are used to explore individual motifs and analyze its advantage. In our experiments, the parameter $k$ in formula (1) is set to 1.5, the neighbor distance $d$ is set to 3 and the count number $c$ is set to 3.

**Table 1:** The number of reconstructions in each species traced by APP2, NeuroGPS-Tree (GPSTree), Neutube and Snake

	Chick	Frog	Fruitfly	Human	Mouse	Silkwoth	Zebrafish	Total
APP2	8	2	91	11	29	7	12	160
GPSTree	8	2	91	11	30	7	12	161
Neutube	8	2	91	9	31	7	13	161
Snake	8	2	91	7	24	7	13	152

### More accurate substructures

The proposed Lamotif algorithm is implemented on the gold166 dataset to explore individual motifs of reconstructions by APP2 [4], Snake [3], Neutube [3] and NeuroGPS-Tree [26]. Individual motifs are successfully generated for most automatic reconstructions (APP2 except 10 neurons, NeuroGPS-Tree except 16 neurons, Neutube except 14 neurons and Snake except 6 neurons). Individual motifs of six neurons are given in Fig. 27, where gold standard and automatic reconstructions are also demonstrated for comparison. In Fig. 28, gold standard reconstructions are in red, automatic reconstructions and their individual motifs are in blue. The number above each column of images is the ID number of the neuron in the dataset. It can be seen that: (1) though APP2, NeuroGPS-Tree, Neutube and Snake are excellent tracing algorithms and generated quite good reconstructions, there are still some inaccurate or redundant substructures. (2) Individual motifs are almost on the gold standard reconstruction and are more accurate than corresponding automatic reconstructions themselves, though they are shorter than the later. That is to say, individual motifs are really some more accurate substructures in automatic reconstructions.

**Fig. 3:**
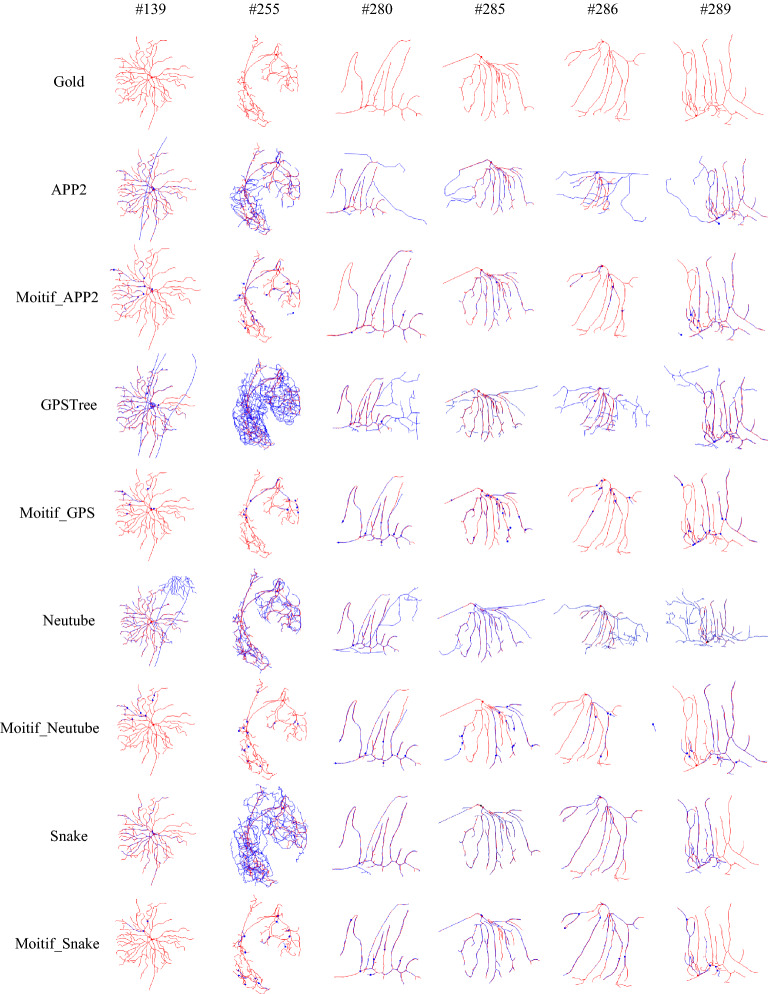
The gold standard (red), APP2, NeuroGPS-Tree, Neutube and Snake reconstructions (blue) of six neurons, and their individual motifs (blue)

To quantitatively evaluate the improvement of individual motifs’ accuracy, we first define a node in a SWC file (individual motifs or automatic reconstructions) as an accurate node if its distance to the nearest ground truth node is no more than 4 voxels [14], and then the precision rate of a reconstruction or its individual motifs is defined as the ratio of the number of accurate nodes to its total node number. For reconstructions from each species generated by each of above four automatic tracing methods, Table 2 gives their average precision rate and that of their corresponding individual motifs. It can be seen that, on all species except frog, individual motifs of all four methods have much higher precision rate than automatic reconstructions. The lower the precision rate of an automatic reconstruction is, the more improvement its individual motifs’ precision rate has. For two frog neurons, automatic reconstructions by all four methods are very accurate with precision rate larger than 95%, and their individual motifs did not improve the precision rate any more. In one word, individual motifs with less substructures are more accurate than automatic reconstructions themselves and have higher reliability.

**Table 2:** Mean precision rates (standard variances) of four kinds of reconstructions (APP2, NeuroGPS-Tree, Neutube and Snake) and their individual motifs on each species neurons in the gold166 dataset

	Chick	Frog	Fruitfly	Human	Mouse	Silkmoth	Zebrafish	Mean (std)
APP2	0.459 ± 0.23	0.993 ± 0.01	0.908 ± 0.18	0.489 ± 0.31	0.855 ± 0.11	0.940 ± 0.03	0.299 ± 0.39	0.804 ± 0.19
Motif_APP2	0.542 ± 0.27	0.993 ± 0.01	0.950 ± 0.10	0.808 ± 0.21	0.906 ± 0.10	0.932 ± 0.03	0.811 ± 0.19	0.901 ± 0.12
GPSTree	0.418 ± 0.16	0.953 ± 0.05	0.815 ± 0.23	0.507 ± 0.30	0.725 ± 0.17	0.718 ± 0.32	0.728 ± 0.16	0.748 ± 0.22
Motif_GPS	0.646 ± 0.32	0.953 ± 0.05	0.924 ± 0.09	0.893 ± 0.07	0.850 ± 0.21	0.907 ± 0.07	0.850 ± 0.07	0.888 ± 0.12
Neutube	0.459 ± 0.15	0.959 ± 0.03	0.905 ± 0.14	0.904 ± 0.11	0.857 ± 0.12	0.919 ± 0.06	0.796 ± 0.08	0.866 ± 0.13
Motif_Neutube	0.649 ± 0.36	0.959 ± 0.03	0.966 ± 0.05	0.978 ± 0.02	0.945 ± 0.08	0.960 ± 0.03	0.949 ± 0.06	0.945 ± 0.07
Snake	0.474 ± 0.20	0.983 ± 0.02	0.776 ± 0.27	0.798 ± 0.06	0.803 ± 0.24	0.911 ± 0.05	0.516 ± 0.29	0.752 ± 0.24
Motif_Snake	0.592 ± 0.31	0.983 ± 0.02	0.905 ± 0.15	0.961 ± 0.05	0.918 ± 0.10	0.950 ± 0.02	0.840 ± 0.16	0.891 ± 0.14

### More accurate automatic reconstructions

In the experiment, Lamotif does not find individual motifs for some automatic reconstructions. The number of APP2, NeuroGPS-Tree, Neutube and Snake reconstructions without individual motifs is 10, 16, 14 and 6, respectively (Table 3). Table 3 also gives the mean precision rate of these reconstructions. Comparing mean precision rates in Table 2 and Table 3, it can be seen that reconstructions without individual motifs have much lower mean precision rates than all reconstructions of each automatic tracing method (0.115 vs 0.804 for APP2, 0.208 vs 0.748 for NeuroGPS-Tree, 0.576 vs 0.866 for Neutube and 0.402 vs 0.752 for Snake).

**Table 3:** Numbers of reconstructions without individual motifs, and their mean precision rates (standard variances)

	APP2	GPSTree	Neutube	Snake
Number	10	16	14	6
Precision	0.115 ± 0.17	0.208 ± 0.26	0.576 ± 0.23	0.402 ± 0.29

**Fig. 4:**
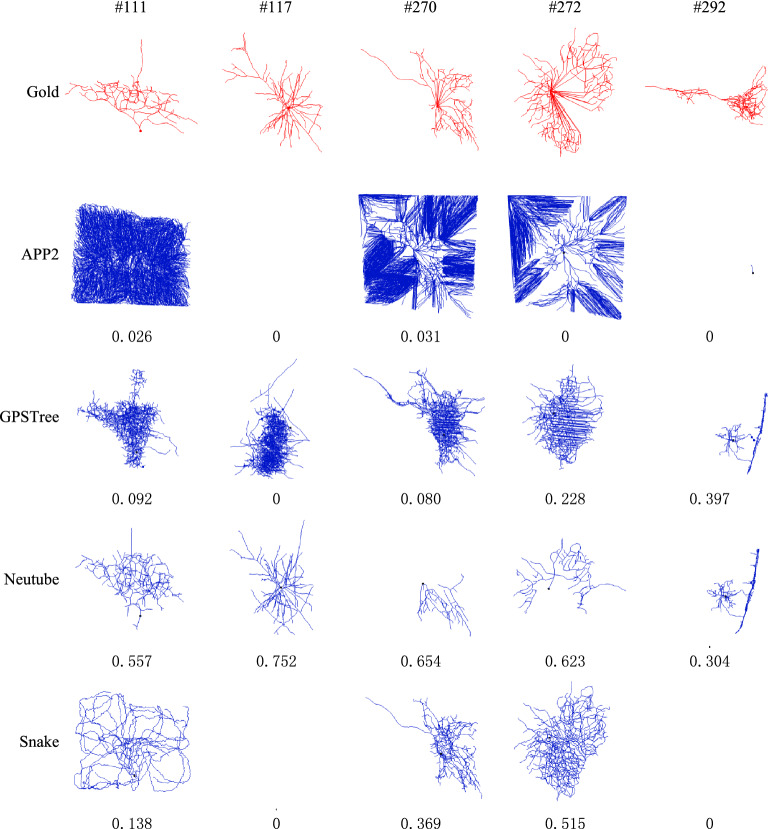
Reconstructions without individual motifs. Numbers in the first line are the ID number of each selected neuron, and the number below each reconstruction is its precision rate

For these reconstructions without individual motifs, there are five neurons which have no individual motifs for any of these four tracing methods. Figure 4 demonstrates the gold standard and four automatic reconstructions of these five neurons. The number below each automatic reconstruction is its precision rate, where 0 means that the tracing algorithm fails to trace the neuron. It can be seen that the morphological structures of these five neurons are quite complex and the performance of most automatic tracing methods is very poor. APP2 fails to trace any meaningful morphological structure or even any neuron signal. NeuroGPS-Tree and Snake lose many (or most) morphological structures and wrongly traces some morphological substructures. The performance of Neutube on these neurons is better than others three methods, and it correctly traces some morphological structures. But due to the bad performance of most other tracing methods, its reconstruction has no overlapping points during voting by all tracing methods, and there is no individual motif. For a neuron, if it has no individual motifs for all automatic tracing methods, its morphological structure is so complex or its microscopic image quality is so poor that most methods fail to trace the structure rightly. In this case, if no gold standard reconstruction has been generated, it is difficult to judge which automatic algorithm does produce more accurate reconstruction. On the other hand, if its reconstruction by some (or all) automatic methods has individual motifs, individual motifs are capable of reflecting the performance of different methods.

**Fig. 5:**
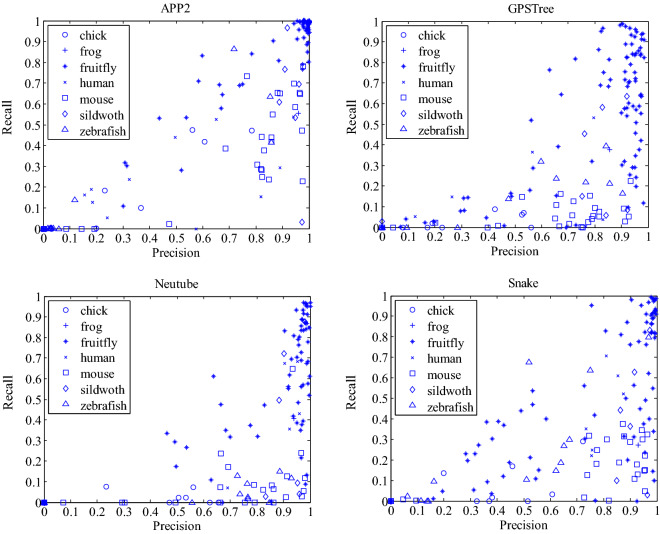
Relationship between recall of individual motifs and precision of reconstructions

In order to further explore the relationship between the quality of a reconstruction and the size of its individual motifs, we define the recall rate of individual motifs as the ratio of the number of nodes in individual motifs to that in its corresponding automatic reconstruction. Note that a node in individual motifs might be an inaccurate node which is not in the gold standard reconstruction. So the above defined recall rate just describes the size of individual motifs compared to its corresponding automatic reconstruction, and it is different from the usual recall rate in information retrieval or patter classification The recall rate of individual motifs for all reconstructions generated by these four automatic tracing methods are calculated and each automatic reconstruction is represented as a point in the precision–recall plane. Figure 5 demonstrates the scatter diagram of APP2, NeuroGPS-Tree, Neutube and Snake reconstructions in gold166 dataset, where reconstructions of neurons from different species are represented by points with different shapes.

From Fig. 5, it can be seen that for these four automatic tracing algorithms, the recall–precision relationship of their reconstructions on neurons from same species have some similarities. For the most abundant (91 from about 161) fruitfly reconstructions, the corresponding points mainly locate in the lower left corner or the upper right corner of the diagram. This means that the recall and precision rate are either small or large at the same time. Therefore, the value of the recall rate can quite accurately reflect the value of the precision rate. It is feasible to use the recall rate of individual motifs to evaluate the quality of automatic reconstruction in fruitfly neuron. Without any gold standard reconstruction, neurons with most accurate automatic reconstructions can be selected just by choosing those with large recall rates. For neurons in other species, their reconstructions by four tracing methods might have larger precision rates but smaller corresponding recall rates. The recall rate of individual motifs is also a candidate index, but there might be some neurons corresponding to reconstructions with large precision rate but small recall rate.

More importantly, for these four algorithms, if their individual motifs have recall rate larger than 0.5, the precision rate of their reconstructions themselves is almost all larger than 0.5, and the basic trend is that the larger the recall rate is, the larger the precision rate is. Of course, a small number of recall rates less than 0.5 corresponds to relatively large precision rates. The recall and precision rate corresponding to APP2 reconstructions shows the strongest linear relationship, and their points is more concentrated in the lower left corner and upper right corner of the diagram. Recall and precision rates of reconstructions by other three algorithms also show a strong linear relationship, especially in fruitfly neurons. In the absence of gold standard reconstruction, the precision rate of an automatic reconstruction cannot be calculated. For reconstructions of different neurons generated by an automatic tracing algorithm, we can roughly judge which reconstruction is more accurate according to the recall rate of its individual motifs. The larger the recall rate of individual motifs is, the more accurate the reconstruction is.

## Conclusions and discussion

For images of a neuron, many reconstructions can be obtained quickly by implementing some automatic tracing methods with different models or assumptions. There are some similar and common substructures in these reconstructions which have high reliability and are called individual motifs. We developed a method called Lamotif to find individual motifs, which is based on local alignment in BlastNeuron. Individual motif can be used without manual checking while constructing a gold standard reconstruction. The performance of an automatic tracing method on some different neurons can be evaluated by the recall rate of their morphological motifs. The larger the recall rate is, the more accurate the reconstruction is. For reconstructions of different neurons in a neuron dataset which are generated by some different automatic tracing methods, reconstructions with larger recall rates are better tracing results. If a neuron contains complex morphological substructures or image quality is very poor, it is difficult to tracing their structures and different tracing methods might generate quite different results. So it might be impossible to find individual motif on this neuron and to find an automatic reconstruction with accurate substructure in these complex regions. Automatic tracing results in these regions should be checked carefully by human experts. In addition, the most time-consuming part of Lamotif is the local alignment algorithm from Blastneuron, and other parts are simple operations like resampling, sorting and calculating distance and so on, which can be quickly done by implementing corresponding plugins in Vaa3D. Its time cost is not high, which is mainly determined by the numbers of automatic reconstructions and the number of nodes in each reconstruction.

Neurons from same category might have similar morphological substructures, and morphological motifs in reconstructions of different neurons might contain some special morphological features of that kind of neurons. Lamotif can be used to find these morphological motifs of different neurons. Though location difference of reconstructions from multiple neurons may be settled by translation, rotation and other pre-processing, morphological motifs of multiple neurons might be very small. Local structures of multiple neurons are quite different and local alignment pairs might be too strict to evaluate them. This results in that Lamotif has not enough local alignment pairs to construct overlap. Some more sophisticated characterization of local similar structure might be the solution of the issue, but it needs to be studied further.

## Data Availability

The dataset used and/or analyzed during the current study are publicly available at the BigNeuron project at https://alleninstitute.org/bigneuron.
